# The Relationship between Energy Poverty and Depression: Focusing on Age as Moderator

**DOI:** 10.1093/geroni/igaf122.2284

**Published:** 2025-12-31

**Authors:** Haeun Kim, Nayeong Gwon, Yeonjung Lee

**Affiliations:** Chung-Ang University, Seoul, Republic of Korea; Chung-Ang University, Seoul, Republic of Korea; Chung-Ang University, Seoul, Republic of Korea

## Abstract

Climate change, including more frequent temperature changes and extreme weather conditions affects this society in many different ways. Given that older adults are disproportionally vulnerable to climate change, it has brought attention to another aspect of poverty, which is energy poverty. Energy poverty (EP) refers to the inability to access and use essential levels of energy for daily living such as heating and air conditioning for cooling. Studies showed that older adults experience higher levels of depression, and they can be more vulnerable to EP than younger counterparts. This study examines the association between EP and depression, and how this relationship will vary by age. Though the suicide rate and poverty among older adults in Korea are extremely high compared to other countries, no studies have paid attention to the potential harmful association between the difficulty in affording energy necessary for daily living and depression. Hierarchical regression analysis and linear prediction were performed using the 2023 Korea Welfare Panel Study (N = 12,986). Results show that those who experienced EP are likely to show higher levels of depression. However, the relationship between EP and depression was different by age. While EP was positively associated with depression for the age group who are 65 and older, the relationship was negative for the younger age group who are under 65. Findings have implications for supporting older adults who lack the necessary energy for daily living, helping to prevent the exacerbation of their depressive symptoms.

